# Primary Squamous Cell Carcinoma of Gallbladder With Hepatic Invasion

**DOI:** 10.7759/cureus.35795

**Published:** 2023-03-05

**Authors:** Conner M Willson, Nathalie Barsoum, Mustafa Tamim Alam Khan, Jennifer Rushton

**Affiliations:** 1 Department of Pathology, Des Moines University, Des Moines, USA; 2 Department of Surgery, University of the Incarnate Word School of Osteopathic Medicine, San Antonio, USA; 3 Department of General Surgery, University of Texas Health Science Center at San Antonio, San Antonio, USA; 4 Department of Pathology, Baptist Health System, San Antonio, USA

**Keywords:** gallbladder pathology, general surgery, hepatic metastasis, surgical oncology, squamous cell carcinoma (scc), cancer gallbladder

## Abstract

Squamous cell carcinoma of the gallbladder is a profoundly rare tumor. It is one of the most aggressive and deadly forms of gallbladder cancer, often being diagnosed at a later stage. There are no definitive risk factors described for this specific type of gallbladder tumor when compared to other forms of gallbladder carcinoma. This case is regarding a 64-year-old female who was found to have primary squamous cell carcinoma of the gallbladder during an attempted cholecystectomy. Her tumor was found to have invaded her liver. The tumor displayed characteristic qualities of a pure squamous cell carcinoma and was positive for CK7 and p63 upon pathological analysis. Best results for treatment for this condition are achieved via R0 resection. Adjuvant therapy with chemoradiation has not been well-defined nor very effective in prior cases.

## Introduction

Squamous cell carcinoma (SCC) of the gallbladder is very rare overall and has a poor prognosis. In 2021, it was reported that the approximate incidence of all gallbladder and biliary tract cancers was 11,980 [1]. SCC is one of the rarest types of gallbladder carcinoma, representing only an estimated 1-4% of all cases [2,3]. In addition, it is considered incredibly aggressive when compared with gallbladder adenocarcinoma, with a reported median survival of seven months after diagnosis [4,5]. As a result of its rarity, extensive knowledge and data regarding this tumor is limited.

SCC of the gallbladder occurs more commonly in females with a median age onset of 65-70 years [3,6]. A majority of the cases reported are in Caucasian individuals, however this may be due to sampling error rather than actual correlation [4,5]. Distinct risk factors for gallbladder SCC have yet to be identified. The only known risk factors are those for all types of gallbladder carcinoma (GBC), which include biliary *Salmonella typhi* infection, gallbladder polyps, chronic cholecystitis, and cholelithiasis [7,8]. Specifically, large cholesterol gallstones have been associated more with SCC rather than adenocarcinoma of the gallbladder in a few cases [9]. One prior report additionally linked *Clonorchis sinensis* infection to SCC of the gallbladder [10]. 

Like most gallbladder carcinomas, SCC is either found early on as incidental findings, or in its later stages, after cholestatic symptoms have manifested. Many of the previous case reports published note concomitant cholelithiasis and/or cholecystitis [11,12]. As a result, around one-fifth of cases are incorrectly diagnosed as a benign condition prior to surgery [5]. Definitive diagnosis is through biopsy, often achieved intraoperatively. On average, SCC presents at a later stage and higher histologic grade when compared to adenocarcinoma of the gallbladder [4-6].

There is no agreed standard for treatment of SCC of the gallbladder, aside from initial surgical resection of the tumor. Due to the aggressiveness of this cancer, extensive invasion and metastasis may prevent full resection and reduce likelihood of survivability. Compared to adenocarcinoma of the gallbladder, SCC typically requires more extensive surgical resection and has more frequent margin positivity. Moreover, survival is worse compared to adenocarcinoma of the gallbladder, even if optimal resection is achieved [12,13]. Adjuvant chemotherapy and radiation have little consensus and data regarding effectiveness is minimal [3]. Chemotherapeutic regimens typically include a combination of gemcitabine and platinum-based, however many other agents have been utilized with varying outcomes [3,12,14]. This regimen is consistent with chemotherapy for other histologic types of gallbladder carcinoma, however SCC has been reported to be less sensitive to chemotherapy, compared to other types of gallbladder carcinoma [15]. There has been some evidence showing that adjuvant radiation therapy can lead to favorable outcomes in patients with SCC of the gallbladder who undergo full surgical resection and negative margins [13,16]. Targeted therapies have not been well-explored due to lack of consensus understanding of drug targets for SCC of the gallbladder [3]. 

Here, we present a case of pure squamous cell carcinoma with hepatic invasion in a 64-year-old female. 

## Case presentation

This is a case of a 64-year-old Caucasian female with a history of morbid obesity, diabetes mellitus, gastroesophageal reflux disease, esophageal stricture, and hypertension who presented to the general surgery clinic for chronic right upper quadrant pain that radiated to her back. The patient reported the pain was mostly post-prandial with exacerbation upon consumption of fat-rich foods. She was referred to general surgery by her primary care physician three months prior to her initial contact with us after an outpatient computed tomography (CT) scan of her abdomen revealed cholelithiasis. In addition to cholelithiasis, this CT scan revealed evidence of cirrhosis, likely due to non-alcoholic steatohepatitis. There was no evidence of any mass on this outpatient CT scan. The patient elected to wait this time before presenting to the surgical clinic as her pain was not severe. One month prior to her clinical evaluation, her pain increased intensely and she lost around 25 lbs as a result. The patient was scheduled for an elective laparoscopic cholecystectomy after the initial clinic visit.

At the time of the surgery, the liver was noted to be enlarged and macronodular, with omentum adhering to its right side, resulting in an inability to visualize the gallbladder. Collateralization of vessels were also present along the falciform ligament and throughout the anterior abdominal wall. Initial dissection of the adherent omentum led to penetration into a cavity. Purulent fluid began oozing from the perforation and cultures of the fluid were obtained. The gallbladder still could not be visualized at this point, and the procedure was converted to an open approach. 

After entry into the right upper quadrant through a Kocher incision, the omentum was dissected and freed off the liver. The gallbladder had appeared to have perforated and the posterior wall appeared to be fibrosis and densely adherent to the gallbladder fossa. Free gallstones were present in the peritoneum. There was difficulty in determining whether the tissue within the gallbladder fossa was omentum or remnant gallbladder wall, and whether or not the abnormal-appearing tissue was granulation tissue or carcinomatosis. Tissue samples from the gallbladder fossa and the liver were sent as frozen sections to pathology, and the intraoperative pathology report was significant for squamous cell carcinoma of both the gallbladder and liver. At this time the procedure was aborted due to risk of uncontrollable hemorrhage. 

**Figure 1:**
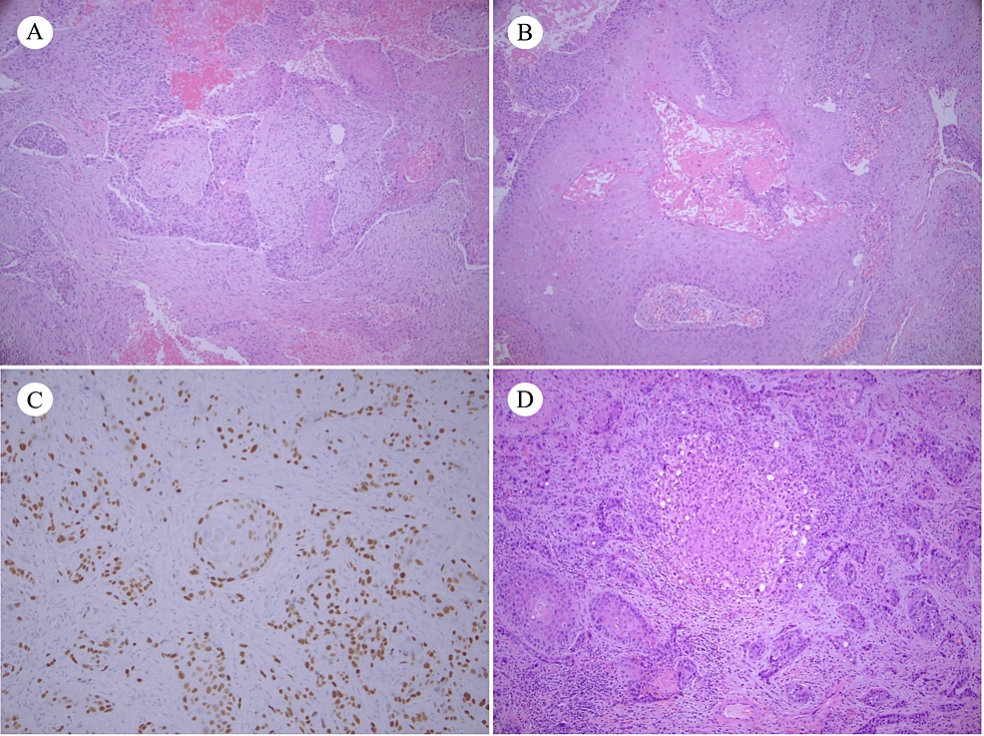
(A) H&E staining of well-differentiated invasive squamous cell carcinoma (SCC) within desmoplastic stroma, with areas of hemorrhage, 10x magnification; (B) H&E staining of keratinizing tumor of gallbladder, 20x magnification; (C) IHC for p63 of primary tumor, revealing squamous differentiation, 20x magnification; (D) H&E of liver metastasis, showing moderate-to-poorly differentiated SCC, 10x magnification.

**Figure 2:**
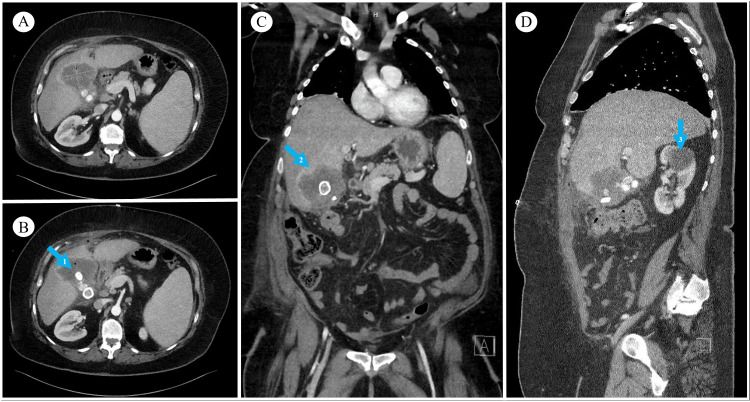
Inpatient CT chest/abdomen/pelvis with IV contrast in axial (A, B), coronal (C), and sagittal views (D). CT images reveal gallstones (arrow 1) and irregular thickening of the gallbladder wall with enhancement and pericholecystic infiltration (arrow 2). Air is present within the gallbladder as well as anterior to the liver. These findings indicate gallbladder carcinoma with hepatic invasion, and cholelithiasis. There is a right renal cyst present (arrow 3), which was incidentally discovered.

The patient was admitted to the hospital for further evaluation. Pathological analysis from tissue within the gallbladder fossa and wall revealed squamous cell carcinoma, with keratinization, necrosis, and fibrous stroma (Figure 1A, 1B). Immunohistochemical analysis of this specimen was positive for tumor protein 63 (p63) and cytokeratin-7 (CK7), both with a similar staining pattern (Figure 1C). The mass was negative for cytokeratin-20 (CK20), GATA binding protein 3 (GATA3), and paired box gene 8 (PAX8). Involved sections of the liver mass also revealed a squamous cell carcinoma with the same features (Figure 1D). CT scans of the chest, abdomen, and pelvis, all with intravenous contrast, were performed on admission and revealed a poorly-defined mass originating from the gallbladder and invading hepatic segments IVb and V (Figure 2). There was no evidence of distant metastatic disease from these CT scans, nor from a nuclear bone scan performed the same day. Her carcinoembryonic antigen (CEA) and carbohydrate antigen 19-9 (CA 19-9) results were within normal limits. She was diagnosed with squamous cell carcinoma of the gallbladder with local hepatic invasion. There were no precursor lesions present. Complete surgical resection was not possible, as the depth of invasion posed a major risk for hemorrhage. After the initial diagnostic workup and recovering from her surgery, she was discharged home and referred to hematology/oncology for further management.

## Discussion

SCC of the gallbladder is exceedingly rare and very aggressive. Overall, the current understanding regarding the epidemiology, pathology, diagnosis, and management of this condition is very limited. Our patient, being an older female with suspected cholecystitis, was a similar presentation to many cases published in current literature [3]. Her largest risk factor was likely chronic cholecystitis due to untreated cholelithiasis. There are no current established methods for screening for this condition, and patients are most often caught in a later stage [4]. Other cases have mentioned associated elevations in CA 19-9 and CEA [12,13]. This patient did not have elevations in either tumor marker.

In order to receive classification as pure SCC of the gallbladder, the tumor must display squamous differentiation without glandular elements. There is a current lack of consistent and consensus classification for defining gallbladder tumors, which may lead to inaccuracies in data reporting for this tumor type [3]. This patient’s tumor consisted of keratinization, intercellular bridges, and fibrous stroma, similar to other previously described cases of SCC of the gallbladder [11,12,17]. On immunohistochemical staining, our patient’s tumor was positive for CK7, which has been reported in a previous case [18]. CK7 is a type II keratin expressed in normal mucosa of the gallbladder. CK7 positivity and CK20 negativity is typical for normal, non-neoplastic gallbladder mucosa. It has been reported to be observed in nearly half of all gallbladder carcinomas, especially when associated with chronic cholecystitis and cholelithiasis [19]. In addition, SCC of the gallbladder with p63 positivity has previously been reported and is seen in our patient’s tumor [20]. p63 is a general marker for squamous epithelium [20].

In regards to treatment of gallbladder SCC, full R0 resection provides the best prognosis [3,4]. Our patient’s tumor was invasive and unresectable at the time of discovery. This tumor is aggressive, yet often clinically silent early on, and many patients present at a stage past that of full resectability [5,6,13]. Treatment after full resection and of unresectable tumors both utilize systemic chemotherapy and radiation [3].

## Conclusions

Pure SCC of the gallbladder is rare, aggressive, and associated with very poor survival rates. Diagnosis at an early stage and R0 resection lead to the best outcomes. Current methods for chemoradiation following surgery and for unresectable tumors are not well-developed nor particularly effective. Further research into the epidemiology, pathology, diagnosis, and treatment of this cancer is necessary to improve our understanding and success with managing this tumor.
